# Reconsideration of Serial Visual Reversal Learning in Octopus *(Octopus vulgaris)* from a Methodological Perspective

**DOI:** 10.3389/fphys.2017.00054

**Published:** 2017-02-07

**Authors:** Alexander Bublitz, Severine R. Weinhold, Sophia Strobel, Guido Dehnhardt, Frederike D. Hanke

**Affiliations:** Institute for Biosciences, Sensory and Cognitive Ecology, University of RostockRostock, Germany

**Keywords:** reversal learning, simultaneous visual discrimination, operant conditioning, behavioral flexibility, secondary reinforcer

## Abstract

Octopuses *(Octopus vulgaris)* are generally considered to possess extraordinary cognitive abilities including the ability to successfully perform in a serial reversal learning task. During reversal learning, an animal is presented with a discrimination problem and after reaching a learning criterion, the signs of the stimuli are reversed: the former positive becomes the negative stimulus and vice versa. If an animal improves its performance over reversals, it is ascribed advanced cognitive abilities. Reversal learning has been tested in octopus in a number of studies. However, the experimental procedures adopted in these studies involved pre-training on the new positive stimulus after a reversal, strong negative reinforcement or might have enabled secondary cueing by the experimenter. These procedures could have all affected the outcome of reversal learning. Thus, in this study, serial visual reversal learning was revisited in octopus. We trained four common octopuses (*O. vulgaris*) to discriminate between 2-dimensional stimuli presented on a monitor in a simultaneous visual discrimination task and reversed the signs of the stimuli each time the animals reached the learning criterion of ≥80% in two consecutive sessions. The animals were trained using operant conditioning techniques including a secondary reinforcer, a rod that was pushed up and down the feeding tube, which signaled the correctness of a response and preceded the subsequent primary reinforcement of food. The experimental protocol did not involve negative reinforcement. One animal completed four reversals and showed progressive improvement, i.e., it decreased its errors to criterion the more reversals it experienced. This animal developed a generalized response strategy. In contrast, another animal completed only one reversal, whereas two animals did not learn to reverse during the first reversal. In conclusion, some octopus individuals can learn to reverse in a visual task demonstrating behavioral flexibility even with a refined methodology.

## Introduction

During reversal learning, an animal has to discriminate between two stimuli. However, after successfully responding to one stimulus with a high performance, the animal has to switch its response pattern because the stimuli will be redefined. The previous positive stimulus (S+), the animal was rewarded for upon choosing, becomes the negative stimulus (S−), and the previous S− becomes the new S+. In a serial reversal learning experiment, the signatures of the stimuli are changed repeatedly every time the animal reaches a specific performance level. The way an animal solves a serial reversal learning experiment tells the experimenter if it has learnt stimulus specific responses or if it has learned to learn (Harlow, 1949; Shettleworth, 1998). The latter would be clear if the animal adopted a win-stay/loose-shift strategy, which could lead to the optimal performance of only one error after a reversal has taken place. By running a reversal learning experiment, behavioral flexibility of a species can be evaluated. Behavioral flexibility is the ability of a species or an individual to develop a new response pattern to unknown stimuli or to alter and adapt an existing response pattern to familiar stimuli. A high degree of flexibility in behavioral response patterns is often required to cope with challenges that animals are confronted with due to environmental changes or unpredictable resources. Behavioral flexibility and the ability to learn more than a mere associate of inhibitory and excitatory reactions to two stimuli as shown when an animal is successful during reversal learning experiments is commonly associated with advanced cognitive abilities (Shettleworth, 1998) beyond mere discrimination learning.

Reversal learning has been studied in numerous vertebrate species including monkeys (Warren, 1966; Milner and Ettlinger, 1970), mice and rats (Mackintosh, 1963; Bissonette and Powell, 2012), cats (Cronholm et al., 1960; Warren, 1966), horses (Fiske and Potter, 1979), kangaroos (Munn, 1964), birds (Bullock and Bitterman, 1962; Gonzalez et al., 1967; Boogert et al., 2010), reptiles (Day et al., 1999; Leal and Powell, 2011), fish (Gonzalez et al., 1967; Parker et al., 2012) and amphibians (Jenkin and Laberge, 2010) among others. In invertebrates, honey bees (Meineke, 1978), crayfish (Capretta and Rea, 1967), cockroaches (Longo, 1964), spiders (Liedtke and Schneider, 2014), and also octopus (Boycott and Young, 1957; Mackintosh, 1964; Young, 1962; Mackintosh and Mackintosh, 1963) have already been confronted with reversal tasks. Experiments on serial reversals in octopus (for overview see Table 1) revealed the ability of the animals to perform multiple reversals (Mackintosh, 1964; Mackintosh and Mackintosh, 1964). In Mackintosh and Mackintosh (1964), the octopods even showed an increase in performance, i.e., the number of errors decreased the more reversals were experienced. This performance compares favorably with a number of vertebrate and invertebrate species tested so far, in rats (Lawrence and Mason, 1955), lizards (Gaalema, 2011), corvids (Bond et al., 2007), pigeons (Gonzalez et al., 1967), isopods (Morrow and Smithson, 1969) as well as bumblebees (Strang and Sherry, 2014). However, in other studies with octopus, no improvement in a series of subsequent reversals could be documented, instead it was found that later reversals took the octopus longer to learn (Mackintosh, 1964; Young, 1962), which compares with the performance of other invertebrates including honey bees (Meineke, 1978) and crayfish (Capretta and Rea, 1967). During reversal learning experiments with octopus, training was often continued for a certain number of trials or sessions after reaching the predefined learning criterion, in order to test whether overtraining had an influence on the reversal learning performance. Mackintosh and Mackintosh (1963) demonstrated in a brightness discrimination task, including a black and white rectangle as stimuli, and by documenting the performance within a single reversal after the acquisition of the original task, that overtrained animals learnt the reversal significantly faster than non-overtrained subjects. However, this phenomenon could only be observed in the presence of irrelevant cues, for instance, the animal could have additionally used either the position or the orientation of the stimuli as an additional cue. Young (1962) investigated repeated reversals in octopuses in a brightness discrimination task, including a black and white circle as stimuli, with the sign of the stimuli being reversed every day for eight reversals without setting any learning criterion. When considering the proportion of errors to trials, performance became progressively worse with repeated reversals. Most likely this was due to a decreasing number of total attacks with subsequent reversals.

**Table 1:** **Overview of the previous visual reversal learning studies including ***Octopus vulgaris*****.

**Reference**	**Focus of the study**	**Stimuli**	**Number of animals**	**Pre-training**	**Electric shock**	**Learning criterion**	**Number of completed reversals**
Boycott and Young, 1957	Reversal of learned responses and effect of vertical lobe removal	Circles, rectangles, L-shaped (Plastic)	9	No	Yes	–	1^+^
Young, 1962	Repeated reversals with a reversal every day comparing performance of animals trained with different stimuli to performance of animals without vertical lobe	Circles, rectangles, squares (Plastic)	26 (in 3 groups) 9 without vertical lobe	No	Yes	–	4–8^#^
Mackintosh, 1964	Effect of overtraining on reversal performance	Rectangles	18 (in 4 groups)	Yes	Yes	80% (in 20 trials)	2–9
Mackintosh and Mackintosh, 1963	Effect of overtraining on reversal performance with and without irrelevant cues	Rectangles (Perspex)	24 (in 3 groups)	Yes	Yes/No	90% (in 20 trials)	1^*^
Mackintosh and Mackintosh, 1964	Reversal learning with and without irrelevant cues (simultaneous stimulus presentation)	Rectangles (Perspex)	10	No	No	80% (in 10 trials)	7–14

+ No classic reversal learning procedure, for details see reference.

# *The signs of the stimuli were reversed every day for nine days without that the performance of the octopuses had reached a specific learning criterion*.

* *Experimenters stopped the reversal training after the first reversal*.

Previous studies on reversal learning in the octopus include some methodological aspects that need to be focused on. First, reversal learning in octopus has only been performed with 3-dimensional stimuli cut mostly from Perspex and fixed to a transparent rod for presentation purposes. They were submerged into the experimental tank probably manually, which might have resulted in the experimenter becoming visible to the experimental subjects. Thus, the experimenter could have provided secondary cues for solving the task. Second, the animals were rewarded with food for a correct response and a response to the S− was often followed by an electric shock. As a consequence, after a reversal, the animals usually had to be pre-trained on the new S+, the former S−, by solely presenting the new S+ for a fixed number of trials or until a certain learning criterion was met (Mackintosh, 1964; Mackintosh and Mackintosh, 1963). This procedure was adopted in order to prevent the animals from stopping to attack directly after a reversal. A cessation of cooperation immediately after a reversal of the experimental animal might happen if, after a reversal, it responded incorrectly because it continued to respond according to the previous definitions of the stimuli, which would ultimately lead to a punishment on the first trial. However, pre-training is considered detrimental in an investigation of learning abilities as the animal learns from every feedback given.

In order to overcome the aforementioned methodological implications, we conducted a visual serial reversal learning experiment with four octopuses as proof of concept for the new methodology and accomplished the following: We presented computer-generated stimuli on monitors and could thus shade the whole aquarium with curtains or carpets in order to avoid secondary cueing by the experimenter. We did not pre-train the animals after a reversal, which was facilitated by using positive reinforcement alone. For reinforcement, we introduced a visual secondary reinforcer, which has never been applied in octopus training before. In conclusion, we could obtain first insight into the serial reversal learning abilities of four octopus individuals with a refined approach.

## Materials and methods

### Ethical statement

This study was carried out in accordance with the directive 2010/63/EU. This study involved a procedure with the severity classification “mild” (Annex VIII). The experiments conducted in this study were approved (6712GH00113) by local authorities (Staatliches Amt für Umwelt und Natur Rostock) according to § 42 of the German law on nature protection. The ARRIVE guidelines checklist (Kilkenny et al., 2010) was the basis for the preparation of this manuscript.

### Experimental subjects

Experimental subjects were four common octopus individuals (*Octopus vulgaris*), four females with a mantle length of 4–8 cm (Table 2), which were subadult at the beginning of the experiment. Three animals were experimentally naïve animals but one, experimental subject Ov3, was already familiar with the experimental procedure and had already received some training in a former visual discrimination task examining concept formation (unpublished data). They were captured in the Mediterranean Sea in the waters of the Tuscan Archipelago, Italy, in spring, and training started with the first phase, feeding by the experimenter (Table 3), as soon as the animal showed interest in food. The animals were kept following the information on maintenance, care, and welfare given for invertebrates in general and cephalopods in particular (Oestmann et al., 1997; Dunlop and King, 2009; Smith et al., 2011; Andrews et al., 2013; Fiorito et al., 2014, 2015). Two subjects, Ov1 and Ov3, were kept in individual 250 l glass tanks (100 × 50 × 50 cm). Subjects Ov2 and Ov4, were housed in a 3000 l sea water aquarium system with individual compartments for the animals (130 × 73 × 86 cm; Table 2). The experiments were conducted in the respective home tanks of the individuals. The tanks were filled with continuously circulating sea water (salinity 33 g/kg, temperature 19–23°C). Artificial illumination was provided mimicking a natural day-night cycle (10/14 h or 12/12 h). To ensure a balanced diet, subjects were given freshly thawed pieces of great northern prawns (*Pandalus borealis*), thawed smelts (*Osmerus eperlanus*), common mussels (*Mytilus edulis*) as well as mussels of the genus *Veneridae* and common shrimps (*Crangon crangon)*. Food was provided to the subjects at least twice a day mainly during the experiments. Individuals were either rewarded with approximately 1 g of northern prawn or mussel per correct response. The type of reward was chosen according to the availability of mussels and to individual preference but was kept constant for one individual over the whole experimental period. Thus, the animals received food according to their performance, which was usually less than 5% of their body weight per day. With a daily food intake of 5% body weight, octopus seems to be fed near satiation (Chapela et al., 2006).

**Table 2:** **Details on the experimental subjects including sex (F female, M male), size as mantle length (in cm), size of the home tank (in l), experimental past, if applicable**.

**Ov**	**Sex**	**Size (cm)**	**Tank size (l)**	**Experimental past**
1	F	5	250	No
2	F	6	800	No
3	F	6	250	Yes
4	F	8	800	No

**Table 3:** **Illustration of the phases of the experiment with procedure and/or the predefined goal of the phase as well as the criterion to end a phase, if applicable**.

**Phases of the experiment**	**Procedure/Goal**	**Criterion**
Training	Taking food from experimenter	
	Establishment of secondary reinforcer by pairing food and secondary reinforcer	
Establishment of experimental procedure	Stationing on the starting position (flower pot)	
	Attacking a moving stimulus (circle) on the right or left side of the monitor	10 attacks on circle/session
	Returning to the feeding tube after a response to the monitor for a reward	
Preference test	Presentation of stimuli planned to be used during reversal training in maximally 10 unrewarded trials; the animal's choices were documented to reveal a possible preference for one or the other stimulus	
Reversal 0 (R0)	Discrimination between the stimuli, stimulus not preferably chosen during the preference test was defined as the S+	Performance ≥ 80 % in 2 sessions of 16-20 trials
Reversal 1 (R1) - and every reversal with uneven number -	Discrimination between the stimuli reversed in sign: new S+ (= S− during R0) and new S− (= S+ during R0)	Performance ≥ 80 % in 2 sessions of 16–20 trials
Reversal 2 (R2) - and every reversal with even number -	Discrimination between the stimuli again reversed in sign: stimuli defined as during R0	Performance ≥ 80 % in 2 sessions of 16–20 trials

Experiments lasted from 30 min up to 2 h, depending on the individual and its motivation. They were carried out 5–7 days a week over a total period of approximately 6 months per individual. The experimental phases (Table 3) followed each other without any large break.

### Experimental setup

**Figure 1:**
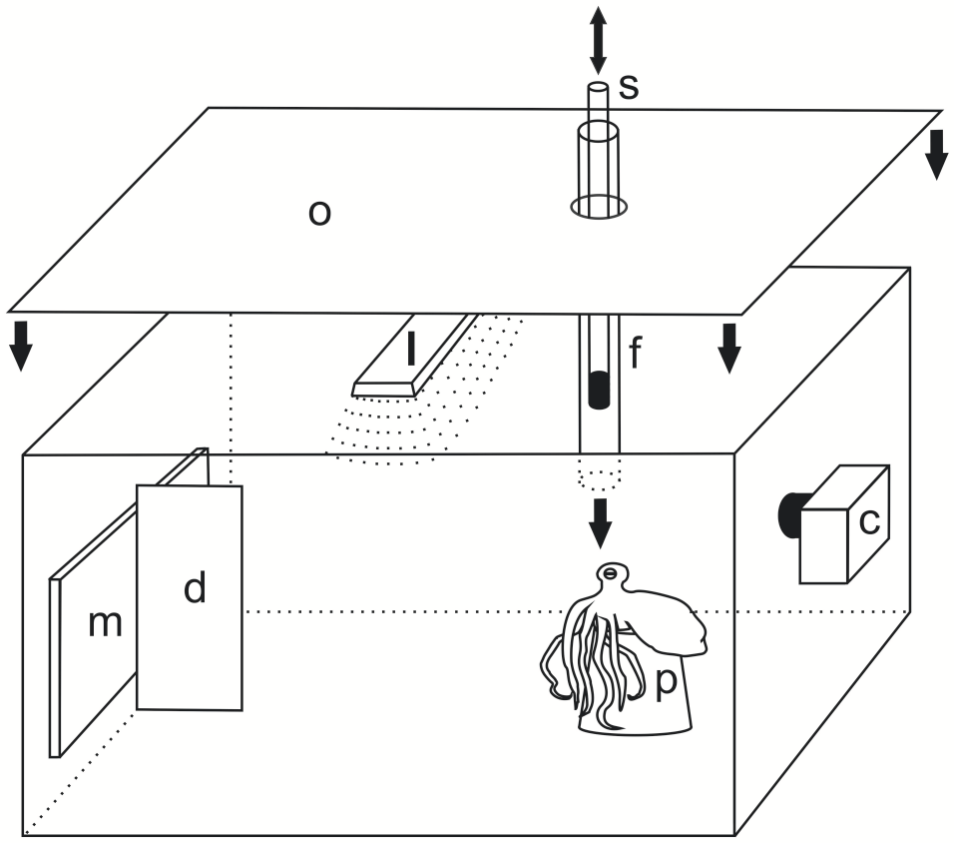
**Experimental setup. Stimuli were presented on a liquid crystal display (LCD) monitor m attached to the tank from outside**. The left and right side of the monitor were separated by a divider **d**. For each single trial, the animal positioned itself on a flower pot **p** at approximately 50 cm distance to the monitor. Reward was provided through a transparent feeding tube **f** which was inserted through the lid of the aquarium. A transparent Perspex rod with a black tip **s** was used as secondary reinforcer. It was inserted into and moved up and down the feeding tube upon a response to the positive stimulus thus indicating a correct choice which was followed by food supply. The whole area was illuminated by a lamp **l**. To avoid secondary cues during experiments, the top of the aquarium as well as the side walls were shielded with an opaque cover **o** (side cover not shown for clarity). Experiments were observed and recorded with the help of a camera **c**. Not drawn to scale.

The general experimental setup is shown in Figure 1. It was installed in the home tank before the arrival of the animal and remained there throughout the experimental period. For stimulus presentation purposes, an LCD monitor was used (21.5 inch, E2251 Full HD, LG electronics, Inc., Seoul, Korea). It was attached to one side wall of the tank from outside. In the middle of the screen, a vertical divider was installed within the tank, which ensured that the animal was giving a precise response either to the left or to the right side of the monitor. Unlike former studies, in which the use of a transparent door kept experimental subjects at a certain distance to the location of stimulus presentation (see e.g., Mackintosh and Mackintosh, 1963; Sutherland and Carr, 1963), a terracotta flower pot was positioned at approximately 50 cm distance to the monitor and was aligned with the center of the monitor. It served as a starting point for each single trial during experiments and ensured that the subjects always had the same viewing angle on the display and the same distance to the stimuli at the beginning of each trial. Close to the flower pot, a transparent acrylic tube (length 55 cm, diameter 3 cm) was inserted through the lid of the aquarium. This tube served to provide the food reward to the subjects. This procedure helped to avoid problems with practicability of food delivery as reported in Boal (1996) and Crancher and King (1972). During experiments, an opaque curtain around the aquarium as well as an opaque cover on the lid of the tank served to keep the experimenter out of sight of the octopus in order to avoid unintentional secondary cueing. The experimenter observed the experimental procedure via a camera (Genius WideCam 1050, KYE System Corporation 2011, Taipei, Taiwan) equipped with a wide angle lens. The whole experimental area was illuminated with a lamp from above.

### Stimuli

**Figure 2:**
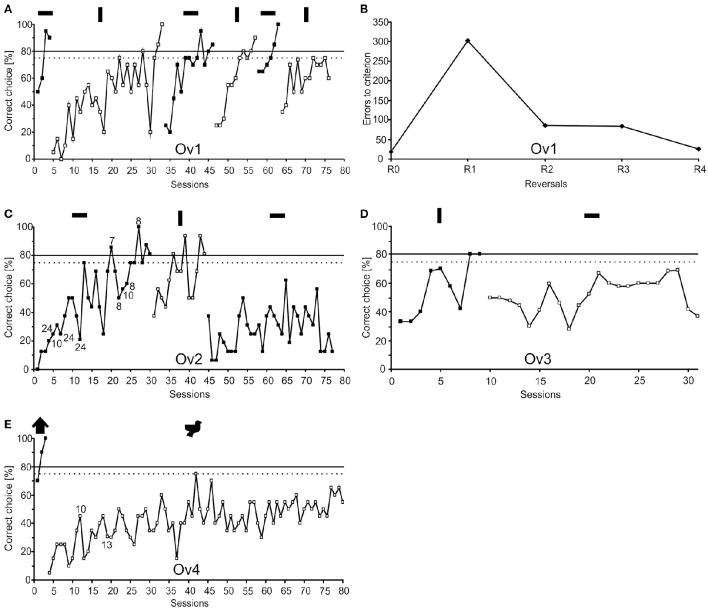
**Learning performance of the four subjects (Ov1–Ov4) together with the stimuli presented to each animal**. The dotted line indicates statistical significance at 75% correct choices (*p* < 0.05). The continuous line indicates the learning criterion of 80% correct choices (for a session of 16 trials: *p* < 0.05, for a session of 20 trials: *p* < 0.01, χ^2^-test) that needed to be met in the sessions of two consecutive days or in 2 sessions of 20 trials. Symbols above the reversals indicate the S+ of the respective phase. (**A)** Learning performance of animal Ov1 during R0 and the five consecutive reversals R1–R5. The number of trials needed to reach the learning criterion decreases with subsequent reversals. (**B)** Number of errors to criterion during R0 and the following completed four reversals in animal Ov1. After an increase of errors in R1 compared to R0, there is a continuously decreasing number of errors to criterion with subsequent reversals. This compares favorably to the performance found in reptiles, birds and mammals. **(C)** Animal Ov2 showed fewer errors in learning R1 compared to R0, but failed to learn R2. With Ov2, a daily session usually consisted of 16 trials; if the session length differed from 16 trials, the number of trials is indicated at the data points. **(D,E)** Animals Ov3 and Ov4 both succeeded in learning R0 but failed to learn R1. Numbers indicate sessions with less than 20 trials.

The stimuli (see inset in Figure 2) used in the experiments were designed with Corel DRAW X5 (Corel Corporation 2012, Ottawa, Canada) and presented to the animals within a Power Point presentation (Microsoft Office 2012, Microsoft Corporation, Redmond USA). All stimuli were presented as black shapes of identical surface area on a gray background on the LCD monitor as this stimulus/background combination elicited attacks by the animals. As an LCD monitor was used for stimulus presentation, octopus, being polarization sensitive (Shashar and Cronin, 1996), might use the polarization and/or luminance contrast for discriminating the stimuli. For all four animals, two different pairs of stimuli were used (Table 4; Figure 2). Three of the animals, Ov1, Ov2, and Ov3 had to discriminate between a vertical and horizontal rectangle (40 × 10 mm) of which two, Ov1 and Ov2, had the horizontal rectangle as S+ in the basic discrimination task (R0) while for subject Ov3 the vertical rectangle was defined as S+ in R0. The rectangles were chosen as octopuses are known to readily discriminate between these stimuli (Sutherland, 1957; Wells, 1978) and they are similar to stimuli used in reversal learning studies in octopus (Boycott and Young, 1957; Mackintosh, 1964). Stimuli were presented to the octopus in a two alternative forced choice experiment. Stimuli were chosen according to the outcome of a preference test with a maximum of 10 unrewarded trials that proceeded reversal training (Tables 3, 4). A preference test was conducted (see Experimental procedure) as octopus has been reported to show pre-existing preferences for some stimuli over others (see e.g., Wells, 1978), which could interfere with learning or reversing in a reversal task. If an animal had a clear preference for one particular stimulus, that stimulus was defined as S−. Subject Ov4 had shown a high preference for the vertical rectangle. To compare the experimental outcome of this animal with the other animal that had also shown a high preference, we switched to a pair of stimuli that revealed no preference to one stimulus over the other, i.e., a bird-like and a house-like shape (both 60 × 60 mm).

**Table 4:** **Overview of the performance of the four octopus individuals during the phases of the reversal learning experiment including the stimuli used during reversal training with the S+ of R0 indicated in brackets, the outcome of the preference test as number of trials, in which the S+ of R0 was chosen out of the total number of preference test trials as well as the number of correct responses per total number of trials to criterion per phase of the reversal training (R0-Rn)**.

**Ov**	**Stimuli**	**Preference test**	**R0**	**R1**	**R2**	**R3**	**R4**	**R5**
1	Rectangle (horizontal)	3/7	59/80	275/580	167/260	130/220	92/120	157/259^*^
2	Rectangle (horizontal)	0/10	217/459	145/224	156/544^*^			
3	Rectangle (vertical)	2/8	98/180	219/440^*^				
4	Bird-House (house)	3/7	51/60	616/1463^*^				

* *Stop of reversal training before learning criterion had been reached*.

The position of the S+ and the S− was pseudo-randomly changed from left to right after Gellermann (1933).

### Experimental procedure

Experiments with each subject were conducted by one experimenter throughout the complete period of training. As soon as the subjects approached the start location, the terracotta flower pot, the trial started by presenting both stimuli on the monitor. After 2 s, they were moved up and down within a range of approximately 3 cm to make the subjects readily attack the stimuli. Subjects were then supposed to respond to the S+ by swimming toward the screen and touching the stimulus within 10 s. The animals were rewarded for each correct response by moving a transparent rod with a black tip, the secondary reinforcer, up and down the feeding tube followed by a piece of food, the primary reinforcer, delivered through the tube. Incorrect choices were followed by directly switching off the stimulus presentation. In case of an inappropriate response i.e., withdrawal from the stimuli or approaching the feeding tube directly without responding, stimuli were switched off after approximately 10 s, and the trial was repeated. Inter-trial interval was limited to 10 min. If the animal did not return to the experiment within these 10 min, the session was ended.

Before initial training could start, all animals had to get used to the general experimental procedure i.e., to approach the start location, to await stimulus presentation, to respond to a stimulus on the monitor and return to the feeding tube and/or start location (Table 3). In order to establish the experimental procedure, only one stimulus was displayed on the monitor, which was a black circle with 4 cm in diameter. Animals were trained until following the experimental procedure for at least 10 times during one session.

Since octopuses have been reported to show pre-existing preferences for some stimuli over others (Boal, 1996), a preference test of maximally 10 unrewarded trials with the respective stimulus pair was performed prior to the training on the discrimination task (Table 3). Sometimes fewer preference trials were conducted (Table 4) as the animals stopped working most likely due to the absence of a reward.

After the preference test, reversal training was started (Table 3). In R0, the experimental subject was asked to respond to the stimulus it had not preferred during preference testing as S+. Subjects performed 16–20 trials a day. These trials were mostly split off into two blocks of 8–10 trials, one block conducted in the morning and one in the afternoon, depending on the individual and its daily motivation. After the animals had reached the learning criterion, predefined as a performance of ≥ 80% correct choices (for a session of 16 trials: *p* < 0.05, for a session of 20 trials: *p* < 0.01, χ^2^-test) in 2 sessions of 16 or 20 trials, the signs of the stimuli were reversed i.e., the former S+ was redefined as S− and the former S− was redefined as the new S+. This experimental stage is referred to as reversal 1 (R1). Apart from this, experimental conditions and procedures remained the same. If subjects again reached the learning criterion in R1, the second reversal (R2) was conducted by redefining the stimuli as in R0. Reversal training continued until experiments had to be stopped because of the (1) animals not responding anymore due to senescence, (2) animals not able to reach the learning criterion in one stage of reversal learning after extensive training or (3) animals' poor motivation during experiments.

### Data analysis

The performance of the individuals was analyzed as the total number of correct choices (in %) summarized for a 16 or 20-trials session. This performance was documented over time for every reversal resulting in classic learning curves (Figure 2). A reversal was considered to be completed if the animal achieved a performance at the preset learning criterion. The learning criterion was predefined with the help of a χ^2^-test to assure that the animal's performance was statistically different from chance performance. For experimental subject Ov1, the number of errors to reach the criterion was additionally analyzed for each reversal separately (Figure 2B). The number of errors to criterion indicated in Figure 2B includes the number of errors made during the 2 sessions required to fulfill the learning criterion.

## Results

All experimental animals were able to discriminate between the given pair of stimuli and successfully completed R0 (Figure 2; Table 4). Ov1 finished the acquisition phase after 4 sessions, Ov2 after 30 sessions, Ov3 after 9 sessions, and Ov4 after 3 sessions. In the reversal training, the performance of the four animals differed in the numbers of completed reversals. Ov1 was able to reach the learning criterion not only in R1 but also in the following three reversals, thus, it successfully finished four consecutive reversals (Figure 2A). Results revealed an increase in errors to criterion in R1 from 21 errors in R0 to 305 errors in R1 (Figure 2B). In contrast, the animal showed a decrease in errors to criterion throughout the reversals following R1 (Figure 2B). However, this animal ceased cooperation during training in R5, most likely due to senescence, and training had to be stopped as a consequence. Ov2 (Figure 2C) finished R1 successfully but in contrast to Ov1, there was a decreasing number of errors during R1 as compared to R0, as only 13 sessions were required to complete R1. In R2, however, the animal did not succeed and training had to be stopped after 33 sessions. Ov3 and Ov4 (Figures 2C,D) reached the learning criterion in R0 within at least 9 sessions, but both animals failed in reaching the learning criterion during Rl. Ov3 failed to rereach the learning criterion in 22 sessions, and training with Ov4 was stopped after 77 sessions.

## Discussion

In this study, four octopus individuals were trained on a serial visual reversal learning experiment as a first proof of concept of the new methodology. From a methodological perspective, this serial reversal learning study stands out from previous discrimination experiments and previous reversal learning experiments in octopus (Boycott and Young, 1957; Mackintosh, 1964; Mackintosh and Mackintosh, 1963; 1964). As a methodological advancement in cephalopod research, a secondary reinforcer, as routinely applied in behavioral experiments with e.g., vertebrates, was introduced in this study to signal the correctness of a response and to announce the subsequent primary reinforcement, food. Our training revealed that the octopus individuals of this and follow-up studies (unpublished data) seem to readily and easily learn the association between food and the secondary reinforcer, they learnt the experimental procedure within a few days, and all individuals acquired the original task. Generally, the use of a secondary reinforcer offers many advantages. First, it allows perfect timing of the feedback after a response as it can instantly signal the correctness which is impossible with food under most circumstances. In previous discrimination experiments, experimenters sometimes attached reinforcement directly to the stimuli in order to avoid a time delay between response and reinforcement (Boal, 1996). However, adopting this procedure most likely enabled the animals to use chemical traces of the food in the water to make their decisions and to improve their performance over time (Boal, 1996). Second, the secondary reinforcer can also function to guide the experimental animal to specific locations such as the starting position, thereby also speeding up experimental procedures as e.g., the animal readily detach from the stimuli upon perceiving the secondary reinforcer. The secondary reinforcer thus substitutes previous handling methods such as chasing the animals. In conclusion, a secondary reinforcer proved to be a useful method for training our octopods in behavioral experiments.

Stimulus presentation was automatized as computer controlled stimuli were presented on monitors (see also Papini and Bitterman, 1991), thus stimulus presentation and movement were very standardized. Moreover, the current type of stimulus presentation allowed shielding the aquarium from all sides prohibiting secondary cueing by the experimenter. In previous octopus discrimination experiments with only a few exceptions (see e.g., Boal, 1993, 1996), stimuli had been manipulated by the experimenter (see e.g., Young, 1956; Muntz et al., 1962; Messenger and Sanders, 1971) and thus, secondary cueing might have affected the results. Generally, secondary cueing is thought to facilitate learning. However, as octopus is easily distracted by extraneous cues, the experimental animals were significantly less successful if the stimuli were submerged and moved by the experimenter (Boal, 1996). In conclusion, the presence of secondary cues is undesirable (Boycott and Young, 1956; reviewed in Boal, 1996). In this study, we provide clear evidence that octopus is able to show learning when stimuli are presented simultaneously and in an automatized fashion without the presence of experimenter given secondary cues.

Unlike previous discrimination experiments involving reversal learning experiments, this study did so without pre-training. Previous reversal learning studies (Mackintosh, 1964; Mackintosh and Mackintosh, 1963) except for Mackintosh and Mackintosh (1964), pre-trained on the new S+ after each reversal. This meant that the animal was presented only with the new S+ and was rewarded upon choosing it for a specific number of sessions or trials (Mackintosh, 1964) or the new S+ was presented until the animal reached a specific performance level (Mackintosh and Mackintosh, 1963). This procedure was adopted as the experimental animal was punished with an electric shock for each incorrect response as well as being reinforced for each correct response. Provided the experimental animal would continue responding to the old S+ although a reversal had taken place, the probability of a mistake in the first trial after a reversal would have been high. As a consequence, many experimental animals directly stopped working. Pre-training seemed to be an appropriate method to overcome this issue. However, already during pre-training, the animal learns about the new S+ which is most likely affecting the results during the subsequent reversal. Moreover, after pre-training on the S+, the animals might only choose on the basis of stimulus familiarity (Boal, 1996). In this study, the experimental subjects were trained with positive reinforcement alone. Therefore, pre-training on the new S+ after a reversal had taken place was unnecessary. Thus, our refined method allowed determining reversal learning abilities in octopus in the classical way without pre-training, which forms the basis for the assessment of learning abilities in octopus and allows interspecific comparison.

Assessing reversal learning abilities with this refined methodology, our results show that at least some octopus individuals can solve a serial visual reversal learning task and can even show progressive improvement. However, the performance was highly individual. Individual performances have already been highlighted for octopus (see e.g., Mather, 1995), even in reversal learning studies (Mackintosh, 1964; Mackintosh and Mackintosh, 1963; 1964). There are many possible reasons that might account for the apparent individuality. First, in line with Young (1956), differences in behavior might be hereditary or due to different experiences in the past. These differences might indeed be pronounced as, due to the fact that it is still not possible to rear octopus in aquaria, wild caught animals have to be taken for experiments. Moreover, cephalopods seem to vary in personality (Mather and Anderson, 1995; Sinn et al., 2006). The personal variability of behaviors along the dimensions activity, reactivity, and avoidance, defined for *Octopus rubescens* (Mather and Anderson, 1995), could, if also applicable for *O. vulgaris*, also lead to different learning performance. In general, a multitude of factors including sex, size, home tank size, or the experimental history (Table 2) might additionally influence the training outcome, this could be a topic for future research.

Secondly, stimulus preferences might affect the individual experimental outcome. The results of Ov1, Ov2, and Ov3 were obtained with a vertical and a horizontal rectangle as the stimuli, which were shown to be easily discriminable by octopus (Boycott and Young, 1956; Sutherland, 1957). The experimental animals of this study showed very strong stimulus preferences as previously reported for a diverse set of stimuli (reviewed in Boal, 1996 and Wells, 1978). Ov1 and Ov2 preferred the vertical rectangle whereas Ov3 mostly responded to the horizontal rectangle during training. The preference for the vertical rectangle could result from the documented preference of octopus to preferably pick the stimulus that is moved along its long axis (Young, 1958, 1965; Sutherland and Muntz, 1959; Sutherland, 1960, 1964; Sutherland and Carr, 1963; Messenger and Sanders, 1972). Strong stimulus preferences could ultimately lead to problems during reversal learning as it might be particularly difficult to learn against a stimulus preference. Whereas, stimulus preferences might thus account for the failure of Ov2 and Ov3 during reversal training, it can, however, not explain why Ov1 was very successful in reversing its response behavior despite its initial strong stimulus preference. A further test was used to elucidate on the effect of the stimuli and of stimulus preferences on reversal learning outcome. Ov4 had shown a high preference for the vertical rectangle and was thus asked to discriminate between a completely different set of stimuli, a house- and bird-like stimulus. With these arbitrarily chosen stimuli, Ov4 almost equally often chose both stimuli in the preference test trials. After a very quick acquisition phase in R0, the experimental animal failed during R1. It is possible that Ov4 had an untrained preference for the house-like stimulus, which was the S+ in R0, which did not become apparent during the few preference test trials, and upon reinforcing in line with the preference, it persisted on responding on the preferred stimulus. Consequently, as already generally discussed in Boal (1996), the performance Ov4 showed in R0 might not have indicated learning as preferences can increase over time in octopus even in the absence of rewards (Fiorito and Scotto, 1992). In conclusion, stimulus preferences might be a factor that strongly influences discrimination experiments. Despite large efforts, stimulus preferences, stimulus processing and discrimination processes, in general, are still poorly understood in octopus.

Thirdly, it is possible that the individual outcome of this study is partially due to the reinforcement type. The animals of the study at hand were only trained using food as positive reinforcement in contrast to previous discrimination experiments in octopus that also used electric shocks as negative reinforcement besides food (see e.g., Sutherland, 1957; exceptions reviewed in Boal, 1996). Food might not be the major factor controlling octopus behavior in its natural environment as octopuses are specializing generalists (Anderson et al., 2008) with an access of available prey (Mather, 1991a). In contrast, octopus is exposed to strong interspecific competition and predator pressure (Alves et al., 2008). Thus, aversive elements might primarily drive decisions in octopus. Indeed one study showed abrupt learning when electric shocks were finally introduced (Sutherland et al., 1963). Electric shocks are very strong aversive elements, however, it is also conceivable to apply mild aversion such as pushing the animal. The role of negative reinforcement in learning discrimination experiments needs further examination.

Fourthly, the experimental design might account for some of the individual variation. We asked the octopus individuals participating in this study to perform in a visual reversal learning experiment. A visual task was chosen due to the octopus' well-developed eyes, its large optic lobes, previous successful visual discrimination experiments including visual reversal learning experiments and its good memory capabilities (Wells, 1978; Mather and Kuba, 2013). An alternative could be to train octopus for a spatial reversal learning task. A more consistent outcome in a spatial task might be expected as spatial orientation is crucial for octopus that occupies dens (Mather, 1991a). They leave their dens for foraging but return later probably navigating via landmarks (Mather, 1991b). From time to time, octopuses also change dens (Mather and O'Dor, 1991), which requires relearning of the spatial layout. There is laboratory evidence from different octopus species that octopuses are capable of spatial learning in detour experiments (Wells, 1964, 1967, 1970), arenas (Boal et al., 2000), and mazes (Walker et al., 1970). Walker et al. (1970) even successfully trained *Octopus maya* to reverse a spatial preference at least once. Good spatial reversal learning abilities have also been demonstrated in a different cephalopod species, the cuttlefish (Karson et al., 2003). Widening the view to other species, most animals tested in visual and spatial reversal learning experiments (see e.g., Holmes and Bitterman, 1966) showed better reversal learning performance with spatial tasks, which further strengthens the hypothesis of better spatial reversal learning abilities, compared to a visual alternative. Current experiments on spatial reversal learning in octopus in our lab will provide deeper insight into reversal learning in octopus.

At least one of the individual octopuses trained in this study with the refined methodology showed good reversal learning performance. Despite our methodology differing from previous studies, Ov1 showed similar performance to the octopus individuals trained in previous reversal learning studies (Table 1; Boycott and Young, 1957; Mackintosh, 1964; Mackintosh and Mackintosh, 1963, 1964). Indeed, Ov1 showed progressive improvement, and it took the animal longer to learn the first reversals than to learn the original task. In contrast, Ov1 made substantially more errors in R1-R3 and stopped cooperating at an earlier stage, during R4. Animals in Mackintosh and Mackintosh (1964) could complete up to 14 reversals, but this was variable between individuals. In the just mentioned study, even one octopus achieved the best possible reversal performance of one error to criterion. In our opinion, these differences in performance can most likely be attributed to methodological differences and individual differences. Generally, the performance Ov1 showed is also comparable to many other organisms including invertebrates and vertebrates. Indeed, many animals such as rats (Mackintosh et al., 1968) and chicken (Bacon et al., 1962) also perform worse during the first reversals as compared to R0. Furthermore, the reversal learning curves suggest that the octopus performance can be explained by proactive interference (Gonzalez et al., 1967; Shettleworth, 1998). At the beginning of R1-R3, Ov1 showed a performance far below chance level, it continued to respond to the S+ as defined during the previous reversal training phase. After a short period, the animal, however, learnt to respond to the new S+. Finally, during R4, Ov1 showed an initial performance at chance level which might indicate that it could no longer remember which stimulus was currently defined as the S+. During R4, the learning curve was very steep before Ov1 stopped cooperating during the fifth reversal, and training was ended. Thus, the best performance of Ov1, 28 errors to criterion, was achieved during R4. Ov1 did not reach the maximum performance possible of one error to criterion seen in other invertebrates such as bumblebees (Chittka, 1998) and cockroaches (Balderrama, 1980). Nevertheless, some octopus individuals seem indeed able to learn to reverse even when the individual is trained to reverse in the “classical” way without pre-training and experimenter given cues. Thus, these octopuses learn more than just stimulus specific responses. Additionally, the results obtained with the octopus individuals in this study provide first evidence that there is no clear separation in reversal learning performance between vertebrates and invertebrates as previously suggested (see e.g., Bitterman, 1965; Warren, 1965) as animals being able to solve reversal tasks even showing progressive improvement can be found in both classes.

The results of Ov1, that showed good reversal learning abilities and even progressive improvement during reversal training, are in line with what we had expected from the octopus biology, adopting an ecological, adaptive approach to learning (Kamil and Mauldin, 1988). Already Young (1961) assumed that long learning phases might be perilous for an octopus when foraging or avoiding predators or conspecifics. Our expectation is based on the fact that the cognitive abilities underlying reversal learning might be generally important for an animal that needs to be behaviorally flexible (Bond et al., 2007). Behavioral flexibility is likely to be important for octopus, living in complex environments that require the animal to respond and adapt quickly to changes in the environment. Furthermore, various features of octopus biology, such as its short life span, active foraging, competition of niches and predator pressure (Packard, 1972; Alves et al., 2008) probably also require the individual to be behaviorally flexible (Mather, 1995; Shettleworth, 1998; Day et al., 1999). An example of a flexible behavior or adaptation to changes in the environment was given by Meisel et al. (2013) who showed that, if a predator is present, octopus switched its activity phase. However, as mentioned, it remains to be answered why only one out of four individuals showed reversal learning abilities consistent with this hypothesis derived from the octopus biology.

In conclusion, with this study, we provide a proof of concept of the new experimental design as all animals learnt the original task and even one individual was able to perform successfully in a reversal learning experiment showing progressive improvement.

## Author contributions

All authors designed the study; AB, SW, and SS trained the octopus; all authors analyzed the data; AB and FH wrote the manuscript; all authors edited the manuscript and approved the final version.

## Funding

This experiment was supported by a grant of the Landesgraduiertenstiftung Mecklenburg-Vorpommern to AB and a grant of the VolkswagenStiftung to GD.

### Conflict of interest statement

The authors declare that the research was conducted in the absence of any commercial or financial relationships that could be construed as a potential conflict of interest.
